# Why vicarious experience is not an instance of synesthesia

**DOI:** 10.3389/fnhum.2013.00128

**Published:** 2013-04-09

**Authors:** Nicolas Rothen, Beat Meier

**Affiliations:** ^1^Department of Psychology, Sackler Centre for Consciousness Science, University of SussexBrighton, UK; ^2^Department of Psychology, Center for Cognition, Learning, and Memory, University of BernBern, Switzerland

A vicarious experience is an empathetic state in response to the observation of others' sensations, emotions, and actions (Keysers and Gazzola, 2009). Vicarious experiences in response to social stimuli are quite common in the general healthy population and they may even constitute an important basis for social behavior. Interestingly, vicarious experiences recruit similar neural processes as the primary experience of a certain sensation, emotion, or action, and it is assumed that the mirror neuron system is involved in these vicarious neural processes (e.g., Morrison et al., 2004; Singer et al., 2004; Jackson et al., 2005).

Synesthesia denotes a condition that leads to specific experiences in response to normal sensory input that is not experienced by non-synesthetes. Synesthetic experiences are characterized as idiosyncratic, involuntarily elicited, and consistent over time (Grossenbacher and Lovelace, 2001; Ward, 2013; but see, Simner, 2011). Synesthesia tends to run in families suggesting a genetic component and has a neural basis (Asher et al., 2006, 2009; Barnett et al., 2008). The best studied and most accepted form of synesthesia is grapheme-color synesthesia. People affected by this type of synesthesia experience colors for numbers and letters printed in black on a white background (e.g., Rothen et al., 2012). On a neural basis, it has been suggested that brain regions concerned with binding processes, the modality of the inducing stimulus, and the modality of the respective sensory experience are involved (e.g., Hubbard et al., 2011; Rouw et al., 2011).

Recently, it has been suggested that also vicarious experiences represent an instance of synesthesia. In particular, the term mirror-sensory synesthesia has been introduced in the scientific literature to describe instances of overt phenomenological experiences reflecting the actual state of an observed sensation and/or emotion (i.e., a phenomenologically overt vicarious experience, Fitzgibbon et al., 2012b). It has been suggested that people who report to have explicit and consciously accessible experiences of touch and/or pain upon the observation of other people being touched or in pain may be called mirror-touch and mirror-pain synesthetes, respectively (Fitzgibbon et al., 2010, 2012b). However, other mirror-sensory forms, such as for example mirror-disgust experiences, seem possible.

Here, we argue that the label synesthesia should be reserved for canonical cases of synesthesia (such as grapheme-color or lexical-gustatory) and we outline similarities and differences between synesthesia and vicarious experiences (Table 1) (for the use of the term synesthesia see also, Deroy and Spence, 2013). By using the term mirrored sensory experiences, we focus on phenomenologically open instances of vicarious experiences because as by the definition of “mirror-sensory synesthesia” phenomenologically less overt forms are not to be regarded as instances of synesthesia.

**Table 1:** **Commonalities and differences between synesthesia and mirrored sensory experiences**.

**Criterion**	**Synesthesia**	**Mirrored sensory experiences**
Prevalence	Rare	Rare to frequent
Developmental trajectory	Early, stable	Late, variable
Neural basis	Specific	General
Bandwith	Typically moderate	Minimal
Consistency	High	Difficult to assess
Idiosyncrasy	High	Minimal
Genetic disposition	Likely and special	Likely but not special
Experience	Conscious	Often conscious

At a first glance, mirrored sensory experiences and synesthesia seem to share many features, but there are also marked differences as already mentioned by Fitzgibbon et al. (2012b). Thus, it is open to debate whether mirrored sensory experiences should be regarded as a form of synesthetic experiences. In order to keep the following critical comparison of the two conditions straightforward, we focus on grapheme-color synesthesia as a well-established form of synesthesia.

For both conditions, the mirrored sensor experience and synesthesia, there is a clear relationship between an inducing stimulus (i.e., inducer in the synesthesia literature) and a resulting concurrent experience (i.e., concurrent in the synesthesia literature). Specifically, this may be the observation of touch for mirrored touch experiences and a letter or number in grapheme-color synesthesia. In both cases the concurrent experiences are triggered automatically and involuntarily. Empirical evidence can be found with variants of the Stroop task (Stroop, 1935), where people experiencing mirrored touch show slower reaction times and more errors in reporting perceived touch for incongruent instances of perceived and observed touch in comparison to congruent instances of perceived and observed touch (Banissy and Ward, 2007). Similarly, grapheme-color synesthetes show slower reaction times in real and synesthetic color naming when presented with graphemes incongruently colored to their experiences in comparison to graphemes congruently colored with their experiences (e.g., Dixon et al., 2004; Ward et al., 2007). However, these kind of Stroop effects do not proof the genuine nature of synesthetic experiences and can be found in non-synesthete controls trained on grapheme-color associations (e.g., Meier and Rothen, 2009; Rothen et al., 2011; Colizoli et al., 2012) or even swimming-style color associations (Rothen et al., 2013a).

Since synesthetic experiences are idiosyncratic and consistent over time, the gold-standard to establish synesthetic experiences is to assess the consistency of the inducer-concurrent relationship in a test-retest procedure. Due to their conscious *experiences*, synesthetes generally exhibit high test-retest consistency for individual inducer-concurrent pairings, but not so controls who have to rely solely on memory (e.g., Baron-Cohen et al., 1987; Eagleman et al., 2007). In contrast, it is not possible to use the test of consistency to mirrored sensory experiences because there is only a minimal bandwidth (i.e., one inducing stimulus such as observed touch or pain for individual forms of mirrored experiences only) and thus, there is no variability in the mirrored experience. Moreover and importantly, the vicarious experience is identical to the inducing stimulus (which is identical to the observed experience).

While the concept of bandwidth (Asher et al., 2006) did not receive much attention in the synesthesia literature, we are not aware of any form of synesthesia which bandwidth is theoretically limited to only one inducer. However, this seems to be the case for the different forms of mirrored sensory experiences (i.e., mirrored touch and mirrored pain). The fact that different levels of intensity of touch or pain, respectively, may or may not induce a mirrored experience has more to do with the associated characteristics of the specific stimulus than actually representing different stimuli (but see, Fitzgibbon et al., 2012b).

The lack of variability (and consequentially lack of categorical organization) in mirrored sensory experiences prevents individual forms of mirrored sensory experiences from sharing with established forms of synesthesia the core criterion of idiosyncratic inducer-concurrent pairings. That is, while A may elicit a red color experience for one grapheme-color synesthete, it may elicit a blue color experience for another grapheme-color synesthete (Grossenbacher and Lovelace, 2001). In contrast, mirrored sensory experiences seem rather systematic than idiosyncratic (for the use of “systematic” in relation to crossmodal correspondences or “weak synesthesia” see, Martino and Marks, 2001). That is, observed soft touch would result in perceived soft touch and observed strong touch would result in perceived strong touch.

The fact that the mirrored sensory experience is identical to the experience of the inducing stimulus constitutes a marked difference between mirrored sensory experiences and established forms of synesthesia, for which the inducer-concurrent relationship is typically somewhat arbitrary and idiosyncratic (but see, Rich et al., 2005; Simner et al., 2005). Due to this characteristic of mirrored sensory experiences, they may be more comparable to the hypothetical form of, for example, grapheme–grapheme synesthesia in which specific graphemes would elicit an experience of the very same grapheme in front of the mind's eye of the perceiver or projected in the space between the inducing grapheme and the eyes of the perceiver—two subtypes that exist in grapheme-color synesthesia described as associator and projector, respectively (Dixon et al., 2004; Ward et al., 2007).

As mentioned earlier, mirrored sensory experiences have a neural basis which is quite different from that of synesthesia. Mirrored experiences are thought to be associated with activity in mirror-neurons which respond not only to an action, sensation, or emotion but also to the observation of the same or a similar action, sensation, or emotion. Mirror-neurons can be found in various different regions of the brain (Keysers and Gazzola, 2009). Mirrored touch and pain experiences are supposed to be associated with activity of mirror-neurons in the somatosensory cortex and the insula (Blakemore et al., 2005; Osborn and Derbyshire, 2010). Hence, mirrored experiences seem to reflect intramodal activity. In contrast, synesthesia seems to reflect explicitly experienced crossmodal activity. That is, synesthesia is associated with brain activity not only related to the inducer but also to the respective specific concurrent (i.e., as if it were sensory in its nature). In grapheme-color synesthesia, these are a grapheme-sensitive region and human color area (hV4) both located in the region of the fusiform gyrus. Moreover, there seem parietal binding mechanisms involved which are thought to underlie the integration of the inducer and concurrent experience (e.g., Rothen et al., 2010; Rouw et al., 2011).

Hence, mirrored experiences seem to be lying on a continuum with vicarious experiences more generally. Indeed, vicarious experiences in the general population are found to activate similar brain areas as mirrored experiences and mirror experiences are found to activate similar brain regions as the respective perceived experience. Collectively, there is increasing evidence for vicarious, mirrored, and real experiences of pain and touch, respectively to have a common neural basis associated with insular, somatosensory, and cingulate cortex activation (e.g., Morrison et al., 2004; Singer et al., 2004; Blakemore et al., 2005; Jackson et al., 2005; Keysers and Gazzola, 2009; Osborn and Derbyshire, 2010). Interestingly, also in non-synesthetes interactions between systems for perceiving and observing touch can be found. That is, sub-threshold stimulation is more likely to be perceived by observing touch to own face than others faces or inanimate objects (Serino et al., 2008). In contrast, the presence/absence of synesthesia seems to reflect a bimodal distribution (Rothen et al., 2013b).

Accordingly, mirrored experiences have been interpreted as the result of an overactive mirror system (Blakemore et al., 2005; Fitzgibbon et al., 2012b). This would be in line with the notion that mirrored experiences are an extreme characteristic of an otherwise normal somatosensory experience on the same continuum (Fitzgibbon et al., 2012b). The relative high incidence (i.e., 30%) of mirrored pain within the general population (Osborn and Derbyshire, 2010) is also suggestive that mirrored experiences are rather normal. In contrast, mirrored touch experiences seem to be more special as the prevalence was estimated to be around 1.6% (Banissy et al., 2009). In addition, an association between enhanced self-rated empathy in people who experience mirrored pain (Osborn and Derbyshire, 2010) and mirrored touch (Banissy and Ward, 2007; but for mirrored pain and empathy in amputees see, Fitzgibbon et al., 2012a) further supports the notion of mirrored sensory experiences as being rather normal experiences on a somatosensory continuum that might be based on empathetic abilities.

Mirrored sensory experiences (i.e., mirrored touch and mirrored pain) seem to be very similar to socially contagious phenomena such as laughter (Provine, 1992), yawning (Provine, 1989; Platek et al., 2003), and itching (Holle et al., 2012). Watching someone laughing can induce a feeling of happiness and put a smile or laugh on the face of the perceiver, watching someone yawning can induce a yawning in the perceiver, and watching someone scratching himself can induce a feeling of itchiness and may lead to the perceiver scratching himself. That is, there is always an inducing stimulus and always a concurrent experience/action. The concurrent experience is elicited automatically, but there is no idiosyncrasy because the concurrent experience is not generally organized in categories. Furthermore, there is also a social component associated with the inducing stimulus (i.e., someone is being perceived doing something) which does not exist for classical forms of synesthesia, but for mirrored sensory experiences. Hence, mirrored sensory experiences may belong to the same category of experiences as socially contagious phenomena which in turn would follow the same continuum as mirrored experiences. Accordingly, it would be interesting to see whether people who are generally more prone to socially contagious phenomena also exhibit higher self-rated empathy (but see, Holle et al., 2012).

Evidence for the similarity between mirrored sensory experiences and socially contagious phenomena can be found on a neural basis. Exactly as mirrored sensory experiences are socially contagious phenomena based on mirror neuron activity and do in fact elicit similar brain activity in the perceiving person as well as the in the observing person (Holle et al., 2012). Accordingly, as used throughout the article, we suggest the terminology “mirrored sensory experience” as a subgroup of socially contagious vicarious phenomena instead of “mirror-sensory synesthesia” as a subgroup of synesthesia.
